# Design of a New 3D Gelatin—Alginate Scaffold Loaded with *Cannabis sativa* Oil

**DOI:** 10.3390/polym14214506

**Published:** 2022-10-25

**Authors:** Pablo Edmundo Antezana, Sofía Municoy, Gorka Orive, Martín Federico Desimone

**Affiliations:** 1 Facultad de Farmacia y Bioquímica, Instituto de Química y Metabolismo del Fármaco (IQUIMEFA), Universidad de Buenos Aires, Consejo Nacional de Investigaciones Científicas y Técnicas (CONICET), Junín 956, Buenos Aires 1113, Argentina; 2 NanoBioCel Research Group, School of Pharmacy, University of the Basque Country (UPV/EHU), 01006 Vitoria-Gasteiz, Spain; 3 Bioaraba, NanoBioCel Research Group, 01009 Vitoria-Gasteiz, Spain; 4 Biomedical Research Networking Centre in Bioengineering, Biomaterials and Nanomedicine (CIBER-BBN), Institute of Health Carlos III, Av Monforte de Lemos 3-5, 28029 Madrid, Spain; 5 University Institute for Regenerative Medicine and Oral Implantology-UIRMI (UPV/EHU-Fundación Eduardo Anitua), 01007 Vitoria-Gasteiz, Spain; 6 Singapore Eye Research Institute, The Academia, 20 College Road, Discovery Tower, Singapore 169856, Singapore

**Keywords:** bioprinting, scaffold, gelatin, alginate, *Cannabis sativa*

## Abstract

There is an increasing medical need for the development of new materials that could replace damaged organs, improve healing of critical wounds or provide the environment required for the formation of a new healthy tissue. The three-dimensional (3D) printing approach has emerged to overcome several of the major deficiencies of tissue engineering. The use of *Cannabis sativa* as a therapy for some diseases has spread throughout the world thanks to its benefits for patients. In this work, we developed a bioink made with gelatin and alginate that was able to be printed using an extrusion 3D bioprinter. The scaffolds obtained were lyophilized, characterized and the swelling was assessed. In addition, the scaffolds were loaded with *Cannabis sativa* oil extract. The presence of the extract provided antimicrobial and antioxidant activity to the 3D scaffolds. Altogether, our results suggest that the new biocompatible material printed with 3D technology and with the addition of *Cannabis sativa* oil could become an attractive alternative to common treatments of soft-tissue infections and wound repair.

## 1. Introduction

There is an increasing medical need for the development of new materials that could replace damaged organs, improve healing of critical wounds or provide the environment required for the formation of a new healthy tissue [1,2]. In addition to the fact that current treatments mean excessive costs [3], chronic or non-healing wounds are related to significant morbidity and mortality rates [4]. In this sense, the need to develop alternative technologies with more efficient treatments for tissue repair remains a global concern. The three-dimensional (3D) printing approach has emerged to overcome several of the major deficiencies of tissue engineering [5]. Using a digital model, 3D-printing technology creates new biocompatible materials with more stable and personalized three-dimensional structures with better mimicking behavior of human organs [6,7,8]. Compared to the conventional skin regeneration techniques, 3D-printed dermal substitutes offer more automation for clinical applications and higher precision for adding living cells and growth factors [9]. To address this, 3D-printing approaches use bioprintable materials, known as bioinks, to manufacture 3D tissue structures with computer-designed geometries. Bioinks are a critical component during the artificial tissue fabrication, and their selection depends on the specific application [10,11]. Although they can be made of synthetic polymers [12], most bioinks are prepared with naturally-derived biomaterials [13,14,15] as they provide a suitable environment for cell adhesion, proliferation, differentiation and the formation of a new tissue. For example, alginate and gelatin are some of the most frequently used bioinks for 3D printing of tissue models due to their high biocompatibility, low cost, good biodegradability, abundance and availability [16,17,18]. Gelatin (GEL) is derived from an acidic or basic partial hydrolysis of collagen, which is a major protein of the natural extracellular matrix [19,20]. Gelatin is especially attractive as a bioink since it has good printability, increases cell adhesion and can be easily degraded by proteases. However, due to its temperature sensitivity and low mechanical strength, gelatin is generally combined with other biomaterials [21,22,23] to improve mechanical properties, enhance printability and increase cell attachment. Alginate (ALG) is a linear anionic polysaccharide with good gelation properties and can be easily solidified with divalent ions (like Ca^2+^, Mg^2+^ and Ba^2+^) without the use of additional chemical cross-linkers [24,25]. In particular, alginate is extensively used to fabricate 3D structures for tissue regeneration, owing to its biocompatibility, low toxicity, biodegradability, low price and simple gelation processes [26]. Similarly to GEL, ALG is usually blended with other polymers to enhance its properties [27]. Therefore, the multicomponent material based on GEL–ALG is an excellent candidate from which to obtain a bioink for the fabrication of 3D printing scaffolds with desired features.

Nowadays, the need to develop innovative materials that could replace and repair damaged tissues leads to the search for multifunctional systems that combine antimicrobial, antioxidant and anti-inflammatory properties with a suitable matrix to stimulate cell proliferation. Particularly, the combination of natural plant extracts with different kinds of dressings is an attractive alternative for wound-healing treatments due to the synergistic action of their bioactive phytochemicals [28,29,30].

*Cannabis sativa* L. is one of the most ancient plants used by humans for medical purposes [31]. Despite the restricted used of *Cannabis sativa* (CS), the medical industry of this annual plant has grown considerably in the last years. The use of Cannabis as a therapy for some diseases or relief of symptoms derived from various treatments has spread throughout the world thanks to the numerous pharmacological effects and benefits for patients [32]. CS (hemp) is an herbaceous dioecious plant of the *Cannabinaceae* family. Its attractive pharmacological properties come from the bioactive phytocannabinoids compounds naturally present in CS [33]. For example, the main components Δ9-tetrahydrocannabinol (THC) and cannabidiol (CBD) interact differentially with the endocannabinoid system in humans, having psychotropic and non-psychotropic activity, respectively [34]. They have been prescribed to deal with depression, sleep disorders and for the treatment of severe diseases like epilepsy, cancer and multiple sclerosis. Moreover, recent works demonstrated the therapeutic potential of CS to improve human skin wound healing [35,36,37]. There is evidence that cannabinoids can regulate cellular and molecular pathways involved in wound restoration, have anti-inflammatory properties, reduce fibrosis and proliferation of hyperproliferative keratinocytes and accelerate injury closure [38]. In addition to this, numerous reports have demonstrated the excellent antibacterial activity of CS, principally against Gram-positive bacteria [39,40]. In this sense, although GEL–ALG 3D-printed scaffolds have been described for different applications [41], to our knowledge this is the first time that the inclusion of *Cannabis sativa* oil extract has been used in a 3D-printed scaffold for skin wound healing.

In this work, a GEL–ALG bioink with good printability properties was prepared to fabricate a tridimensional printed scaffold for wound healing. The biocompatible material was loaded with *Cannabis sativa* oil extract to obtain a 3D scaffold with antimicrobial and antioxidant activity. Altogether, these results confirm the development of a new biocompatible material printed with 3D technology and functionalized with *Cannabis sativa* oil, leading to a promising alternative for common treatments of wound infections and for wound-healing applications.

## 2. Materials and Methods

### 2.1. Synthesis of Gelatin and Alginate Bioink

Type A gelatin from porcine skin (Sigma Aldrich, St. Louis, MO, USA) and low-viscosity sodium alginate derived from brown algae (Sigma Aldrich) were used. Different preparation procedures were evaluated, such as the temperature and the stirring time, until the best set-up conditions were achieved. The bioink was made by mixing gelatin 0.7 g and alginate 0.3 g in 10 mL of PBS.

### 2.2. Bioprinting Parameters

The bioink was printed using a Bio X extrusion-based 3D bioprinter from Cellink (San Carlos, CA, USA). Through a 27 G conical nozzle, 20 mm × 20 mm square grid-shaped scaffolds were printed. Heart OSTM software was used for printing; and the model was designed using the OBJ Viewer designing program. The bioinks were printed on the 3D bioprinter, in which the optimal printing parameters were determined and were subsequently used with all the prints (170 kPa pressure and 27 °C temperature).

### 2.3. Rheological Properties

First, amplitude sweeps were performed to determine the linear viscoelastic range. The elastic and the viscous moduli, G’ and G”, respectively, were determined by small-amplitude oscillatory shear flow experiments, using a rheometer AR2000 (TA instruments, New Castle, DE, USA). For this, parallel plates with a diameter of 20 mm were used and the response of the material in a 0.001–10 Hz range of frequency and tension 1% were evaluated at 27 °C

### 2.4. Cross-Linking of the GEL–ALG Scaffolds

The scaffolds obtained by bioprinting were immersed in a CaCl_2_ solution to carry out the cross-linking procedure. Different concentrations of CaCl_2_ were evaluated until the optimum of 100 mM was achieved.

### 2.5. GEL–ALG Scaffolds Characterization

#### 2.5.1. GEL–ALG Scaffolds Lyophilization

After cross-linking and gelation, the scaffolds were subjected to a lyophilization process for 42 h using a Telstar freeze-dryer (Lyobeta, Tarrasa, Spain). For this, the following drying cycle was programmed: freezing for 3 h, primary drying for 12 h and secondary drying for 24 h. The pressure was adjusted to 0.2 mBar during primary drying. Table 1 summarizes the exact process parameters.

#### 2.5.2. GEL–ALG Scaffolds Swelling

The scaffolds were dry-weighed, then impregnated with PBS and finally, after determining that all the PBS was absorbed, they were weighed again. In order to quantify the absorption capacity of the gel, the following calculation was performed:

$$\mathrm{Swelling}\;\left(\%\right)=\frac{W_{\mathrm{wet}}-W_{\mathrm{dried}}}{W_{\mathrm{dried}}}\times 100$$

#### 2.5.3. GEL–ALG Scaffolds Scanning Electron Microscopy (SEM)

For morphological characterization, samples were lyophilized as indicated in Section 2.5.1. Cross-sections of the lyophilized film samples were then cut using a cold scalpel. Finally, the samples were examined with a Jeol JSM-5400 scanning electron microscope (JEOL, Tokyo, Japan).

#### 2.5.4. Cytotoxicity Experiments

3T3 fibroblasts were used to study the biocompatibility of the scaffolds. The cells were cultured in a humidified chamber (95% air; 5% CO_2_) at 37 °C in Dulbecco’s Modified Eagle’s Medium (DMEM) and supplemented with 10% fetal calf serum and 1% penicillin–streptomycin. Once in confluence, cells were trypsinized and counted by a Neubauer camera. On top of each scaffold, 1 × 10^4^ cells were seeded with 1000 µL of DMEM and were incubated at 37 °C for 24 and 48 h. The colorimetric 3-(4,5-dimethyl-thiazol-2-yl)-2,5-diphenyl-tetrazolium bromide (MTT) assay was used to evaluate the GEL–ALG scaffold biocompatibility. After 24 or 48 h, we removed the medium, added 0.5 mL of MTT solution (5 mg/mL) and incubated for 3 h at 37 °C. Then the MTT solution was discarded and 0.5 mL of absolute ethanol was added after washing the scaffolds with PBS 1X. The absorbance values were measured at 570 nm and results are expressed as mean ± SD from triplicate experiments.

### 2.6. Addition of Cannabis sativa Oil to GEL–ALG Scaffold (GEL–ALG–CS Scaffold)

To prepare the GEL–ALG–CS scaffolds, 400 µL of *Cannabis sativa* oil extract were added to the GEL–ALG scaffolds for 24 h. *Cannabis sativa* oil possesses: 0.066 mg/mL cannabidiol (CBD), 0.036 mg/mL cannabidiol acid (CBDA), 0.030 mg/mL cannabinol (CBN), 1.267 mg/mL ∆9-tetrahydrocannabinol (THC) and 0.226 tetrahidrocannabinol acid (THCA), determined by HPLC-UV (high performance liquid chromatography with spectroscopy UV detector). For HPLC analysis, samples were diluted and extracted with methanol (HPLC-quality). Standard solutions of HPLC-quality cannabinoids (Cerilliant) were used and ibuprofen SCBT was used as internal standard. Chromatograms were obtained using an Agilent Technologies, CA, USA 1200 Series HPLC with a Diode Array Detector and a C18 column. The mobile phase was composed of acetonitrile (HPLC quality) and formate buffer solution (Biopack). This method, HPLC-UV, was used to determine the release of the different components of *the Cannabis sativa* oil extract during time.

#### 2.6.1. Fourier Transform Infrared (FTIR) Analysis

FTIR spectra of *Cannabis sativa* oil extract, GEL–ALG scaffold and GEL–ALG–CS scaffold were obtained over the range of 4000–500 cm^−1^, using a FTIR-Raman Nicolet iS 50 (Thermo Scientific, Waltham, MA, USA). Samples was dried under a nitrogen flow and the powder was then placed on the attenuated total reflection accessory of the spectrometer [42].

#### 2.6.2. Scaffold Enzymatic Degradation

We assess the scaffolds’ degradation by collagenase digestion, incubating GEL–ALG scaffold and GEL–ALG–CS scaffolds with collagenase enzyme solution in PBS at 37 °C (1 mL of a 10 U/mL solution of type I collagenase Gibco^®^, 260 U/mg). Scaffold’s weights were measured at different time intervals [43].

#### 2.6.3. Antioxidant Capacity

To study the antioxidant activity of the scaffolds, DPPH colorimetric assay was performed [29]. This method is based on the measurement of the scavenging activity of the 2,2-diphenyl-1-picrylhydrazyl free radical (DPPH•). To study the antioxidant activity of *Cannabis sativa*, 3 mL of a methanolic solution of DPPH• (25 mg/L) were added to 200 μL of the CS extract. For GEL–ALG and GEL–ALG–CS, the same volume of DPPH• (25 mg/L) was added to the scaffolds and incubated for 5 min or 10 min at room temperature. In addition, two GEL–ALG–CS scaffolds with different weights were evaluated. The absorbances were evaluated at 517 nm. The antioxidant capacity of CS, GEL–ALG and GEL–ALG–CS was calculated as percentage of inhibition with the following equation:

%inhibition = [1 − (Abssample/AbsDPPH solution)] × 100

#### 2.6.4. Antimicrobial Activity

We evaluated the bactericidal activity of GEL–ALG scaffold and GEL–ALG–CS scaffold on Gram-positive (*Staphylococcus aureus*) and Gram-negative (*Escherichia coli*) bacteria. To reach the optimal growth conditions of *S. aureus* (ATCC 29213) and *E. coli* (ATCC 9637), they were incubated overnight at 37 °C in tryptic soy agar (TSA) medium (enzymatic digest of casein 15 g/L, enzymatic digest of soybean 5 g/L, NaCl 5/L, agar 15 g/L). Then, they were diluted to obtain 1 × 10^6^ Colony Forming Units (CFU) per mL. The antimicrobial activity was evaluated using two methods: the solid and the dilution method. For the disk diffusion method, 20 µL of a bacterial suspension 1:1000 was spread on an agar Petri dish. Then, GEL–ALG and GEL–ALG–CS scaffolds were placed on the agar surface and incubated at 37 °C. The inhibitory action of the scaffolds was determined after 24 h by measuring the diameter of the bacteria-free areas surrounding the scaffolds.

On the other hand, to study the antimicrobial capacity of the scaffolds by the dilution method, 100 µL of the bacterial suspension 1:1000 was mixed with *Cannabis sativa* oil extract and GEL–ALG scaffold and GEL–ALG–CS scaffolds with 900 µL of Luria Bertani (LB) medium (yeast extract, 5 g/L; NaCl, 10 g/L; and tryptone, 10 g/L). The samples were incubated at 37 °C for 24 h. After that, serial dilutions from the supernatants were made in PBS. Aliquots of 20 µL of the dilutions were seeded on agar Petri dishes and maintained at 37 °C for 12 h. CFU were determined by manually counting the colonies on the plate. Results are expressed as mean ± SD from triplicate experiments.

#### 2.6.5. Biocompatibility

Biocompatibility was evaluated in 3T3 fibroblasts, as previously described in Section 2.5.4, using MTT assay.

### 2.7. Sterilization

All the scaffolds were sterilized for 30 min using UV-C light, since it has germicidal activity [44]. The sterilization was confirmed using an adapted method from the European Pharmacopoeia: in brief, the sterilized scaffold was incubated in LB at 37 °C for 7 days and cultivated in a petri dish. No growth was observed after the 7 days evaluated.

### 2.8. Statistical Analysis

Statistical analysis was performed using the statistical software GraphPad Prism 5.0 software (GraphPad Software, La Jolla, CA, USA). Data were expressed as mean ± SD of triplicate experiments. The differences were analyzed using one-way ANOVA, followed by the Tuckey’s post-test; *p* < 0.05 was considered significant.

## 3. Results and Discussion

### 3.1. Synthesis and Characterization of Gelatin and Alginate Bioink

In order to develop a good, printable bioink, it is essential to determine the best preparation process. In this sense, we probed three different procedures by mixing the gelatin and alginate in three different conditions, as is shown in Table 2.

**Figure 1:**
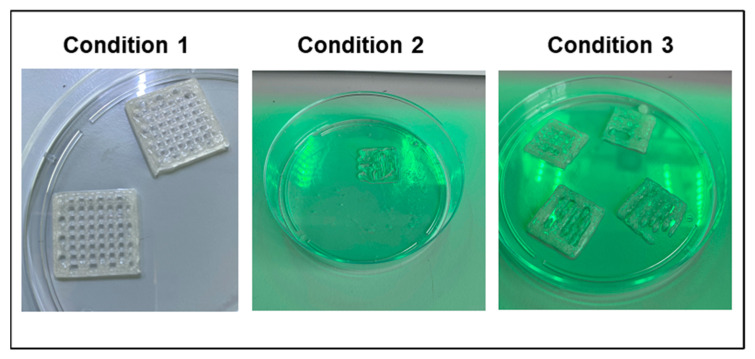
GEL–ALG scaffolds printed under the three conditions.

During the bioprinting process an extrusion method was used, as described in the Materials and Methods section. We used pneumatic pressure to extrude the ink from the needle in an uninterrupted line [45]. After the bioprinting process, we found that inks prepared under Conditions 2 and 3 were not able to be printed. On the other hand, Condition 1 allowed us to prepare an ink that was printed successfully (Figure 1).

Therefore, the first step to prepare the bioink was to mix gelatin and alginate, then to add the PBS and finally to shake at 60 °C for 2 h. In the case of Condition 2, it is possible that the lack of temperature affected the formation of a homogeneous mixture. This could be probably due to the fact that gelatin is sensitive to temperature and its ability to generate structures is determined by temperature [46]. On the other hand, it is possible that in Condition 3 the problem was in the dissolution of the alginate. This is due to the fact that alginate requires immediate and constant agitation from the beginning of its dissolution in order to obtain a homogeneous consistency and make an accurate printing [47,48].

**Figure 2:**
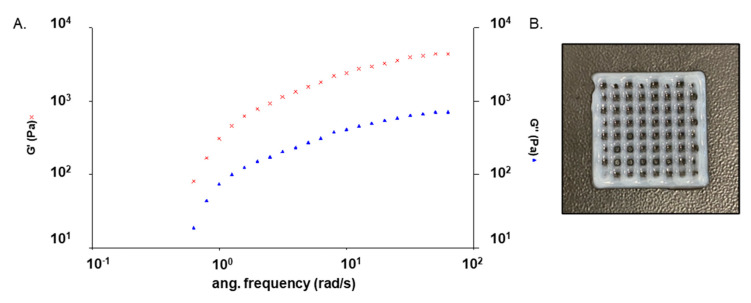
(**A**) Variation of the elastic (G’, red crosses) and viscous modulus (G”, blue triangles) with frequency of GEL–ALG ink. (**B**) GEL–ALG scaffold photograph after printing.

We evaluated the mechanical properties and stability of GEL–ALG bioink by studying its rheological behavior at 25° C. This test determines whether the bioink has a predominantly elastic or viscous behavior. G’ corresponds to the elastic component, while G” corresponds to the viscous one. The relationship of the two modules provides information on the characteristics of the sample. It is widely known that any type of material has a solid component and a liquid component. Depending on the predominant component in each case, we can classify the materials as viscoelastic solids or viscoelastic liquids. This step is crucial for the success of clinical applications while developing a new biomaterial. As it is shown in Figure 2A, the ink possesses a gel-like behavior, since the values of G’ elastic components exceed the values of G” viscous components. Figure 2B displays a photograph of the GEL–ALG scaffold after printing, showing that the bioink was printed with precision.

**Figure 3:**
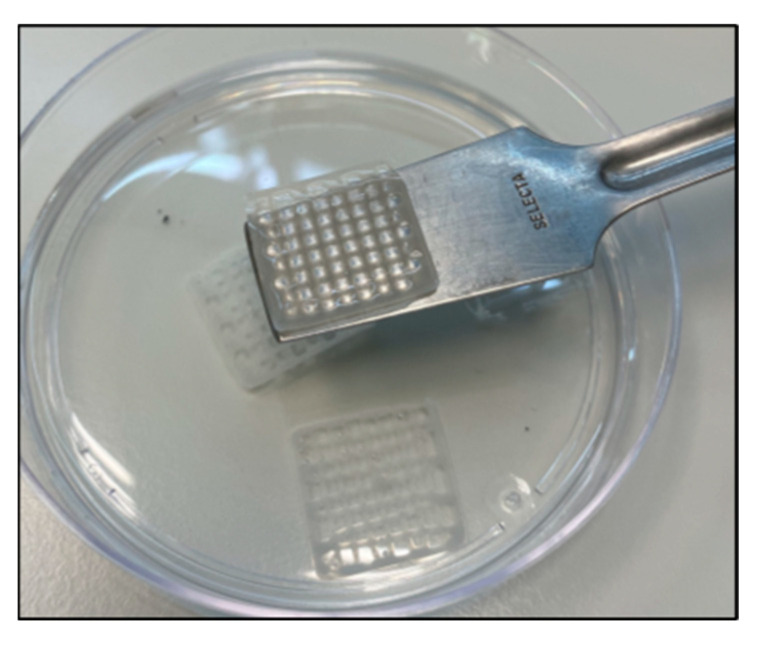
GEL–ALG scaffolds photograph after cross-linking with CaCl_2_.

Once the scaffolds had been printed, we proceeded with the cross-linking. The bioprinted GEL–ALG scaffolds were cross-linked with 100 mM CaCl_2_ (Figure 3). The cross-linking allows an improvement in the structure of the scaffold, leading to a decrease in its degradability and an increase in its stability [49]. In addition, the use of calcium salt, which is an economical compound, is favorable when considering production on larger scales.

**Figure 4:**
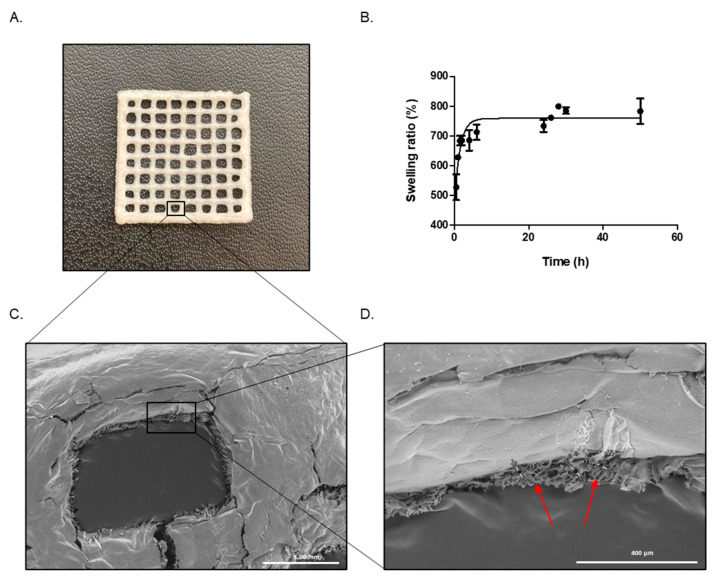
(**A**) Lyophilized GEL–ALG scaffold photograph. (**B**) GEL–ALG scaffold swelling determination. Values represent mean ± SD. (**C**) Scanning Electron Microscopy (SEM) of GEL–ALG scaffold. (**D**) Close-up of the porous area observed in image (**C**).

After achieving a printable bioink, we decided to lyophilize the GEL–ALG scaffolds. After this procedure, it was interesting to observe that the scaffolds maintained their structure and dimensions (Figure 4A). As a result of this, it can be concluded that the scaffolds could be printed, lyophilized and then, at the time of use, rehydrated and/or impregnated with therapeutic molecules. This would allow a multifunctional action for its dermal application.

Another important feature to be characterized is the swelling capacity of the scaffold. In this sense, swelling is important for a biomedical material because it is related to the exchange of substances as well as other properties, such as mechanical properties or flexibility [50]. We found that GEL–ALG scaffolds reach the maximum PBS uptake at 6 h, remaining constant for more than 50 h. In addition, the GEL–ALG scaffolds reached 800% of swelling (Figure 4B).

The next step was to study the structure of the scaffolds through SEM. The use of this microscopy is interesting, since it is a technique that allows a wide variety of materials to be characterized both at macro- and microscale; among these can be mentioned nano-structured materials, metal alloys, polymers, minerals, fibers, thin films and biomaterials [51]. It is essential to know the internal and external structure of the scaffolds, since this has been recognized as an important factor in defining cell behavior [52]. When analyzing scaffolds by SEM, a compact and smooth surface was observed (Figure 4C) whose porosity could be appreciated when making a cross section (Figure 4D). The size of these pores was determined, confirming that it is a highly porous material with an average pore size of 15.0 ± 2.3 µm.

In this sense, the main function of a scaffold based on biodegradable polymers is that it must be capable of supporting the adhesion, proliferation and differentiation of cells until the regeneration of tissues at an injured site. For this reason, the porosity of the scaffolds is important, since some pore sizes can improve cell activity. The optimal size and geometry are highly dependent on the specific cell type growing at the injured sites [53]. Studies in cells have shown that fibroblasts have a bridging mechanism that allows them to extend themselves to fill pores of different sizes, even the largest ones (from 5 to 90 mm). On the other hand, since endothelial cells do not have this bridging system, they prefer pores with sizes closer to their own dimensions [54]. Thus, the porosity could serve as an ideal structure to allow colonization by cells through infiltration and cell growth. It is worth mentioning that a high porosity also provides a favorable structure to allow the incorporation of different therapeutic molecules. Therefore, these scaffolds are extremely interesting for their application in future perspectives.

**Figure 5:**
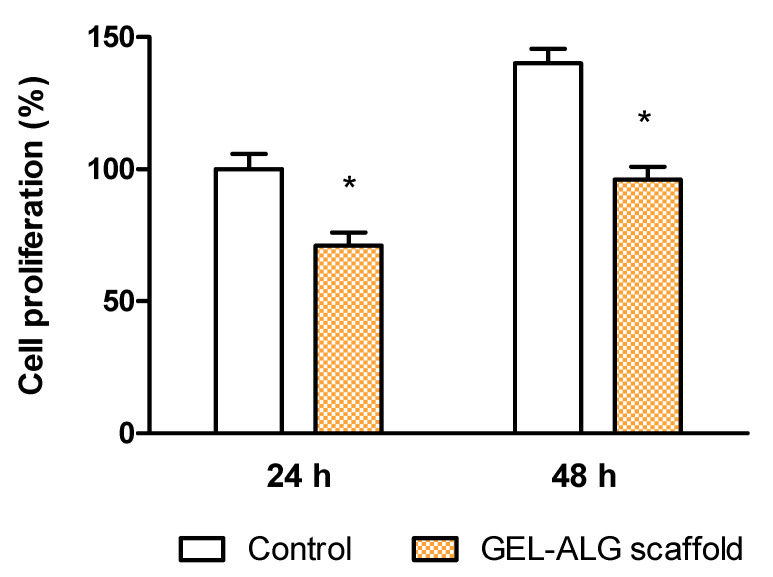
Viability of 3T3 fibroblasts evaluated by the MTT test at 24 h for control and GEL–ALG scaffold. Viability rate in control was considered as 100%. Results are expressed as mean ± SD from triplicate experiments. * *p* < 0.05.

It is widely reported that both gelatin and alginate are biocompatible and used for various biomedical applications [55,56]. Given that 3D-printed GEL–ALG scaffold could be applied to promote would healing, its cytotoxicity was also evaluated. Herein, a fibroblast cell line was chosen as a model cell due to its main role in skin wound reparation. For this, a 3T3 fibroblast cell culture was incubated in DMEM in the presence of the scaffolds, at 37 °C in a humidity incubator. After 24 h, cell viability was determined by the in vitro MTT assay. As it was expected, when fibroblasts were exposed to the GEL–ALG tridimensional material, a 70% cell viability was reached compared with the cell control (Figure 5). This demonstrates the good biocompatibility of the combined bioink and its potential use as a biomaterial for wound healing. In order to evaluate if the GEL–ALG scaffold affected the cell viability during time, we studied the cytotoxicity at 48 h. Figure 5 shows that after that time the cells continued growing when exposed to the GEL–ALG scaffold.

### 3.2. Addition of Cannabis sativa Oil to GEL–ALG Scaffold (GEL–ALG–CS Scaffold)

**Figure 6:**
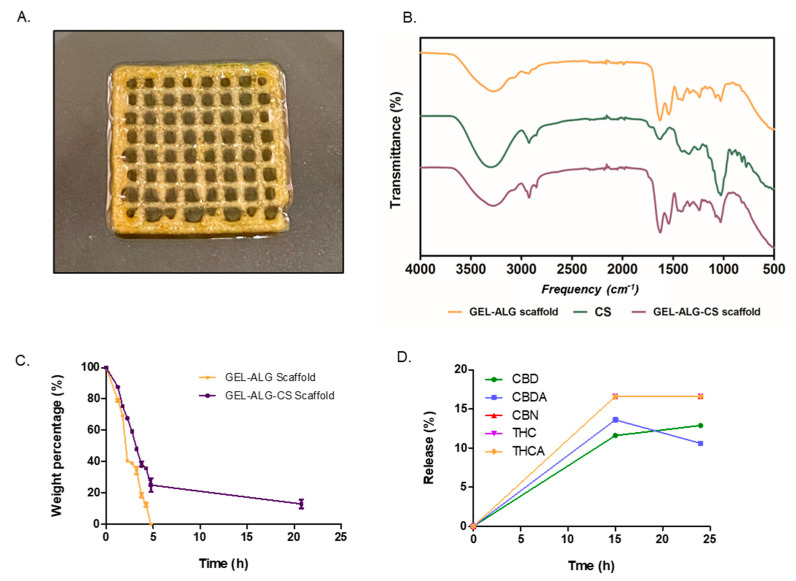
(**A**) Photograph of GEL–ALG scaffold loaded with *Cannabis sativa* oil. (**B**) FTIR (**C**) Metalloproteinase degradation of scaffolds. Variation of weight percentage with time is shown for GEL–ALG scaffold (orange) and GEL–ALG–CS scaffold (violet). (**D**) Release of *Cannabis sativa* oil extract. Results are expressed as mean ± SD from triplicate experiments.

Due to the interesting properties displayed by the GEL–ALG scaffold, we decided to add *Cannabis sativa* oil to the scaffold, leading to a concentration of 0.55 mg CBD/g in the scaffold (Figure 6A). The aim was to obtain a new biomaterial with antioxidant and antimicrobial properties [36]. To confirm that CS was successfully incorporated into the scaffold, an FTIR analysis was performed. Figure 6B shows FTIR spectra of GEL–ALG scaffolds, CS extract and GEL–ALG scaffolds containing CS (GEL–ALG–CS). GEL–ALG scaffolds show characteristics peaks within the mixture that demonstrate a strong intermolecular attraction between both macromolecules through electrostatic interactions [57]. GEL–ALG spectrum shows a broad band at 3272 cm^−^^1^, assigned to NH and OH stretching vibrations of amides, and a peak at 1641 cm^−^^1^ that corresponds to the coupling of C=O and CN stretching vibrations of amide I of gelatin [58] with the asymmetric stretching vibrations of COO- of alginate [59]. The peak at 1542 cm^−^^1^ is assigned to NH and CN vibration of groups in amide II of gelatin [60], while the signals at 1031 and 1079 cm^−^^1^ are attributed to vibration of C-O and CO-C groups in mannuronic and guluronic units of alginate [61], respectively. The broad peak observed at 3308 cm^−^^1^ in the FTIR spectrum of CS represents the OH stretching vibrational band and the signals at 2925 and 2875 cm^−^^1^ correspond to the symmetric and asymmetric stretching vibrations of CH alkane groups. Furthermore, the absorbance peaks at 1637 and 1049 cm^−^^1^ are attributed to C=C double-bond stretching and C-O stretching of CS, respectively. Incorporation of CS in GEL–ALG scaffolds was confirmed by analyzing the GEL–ALG–CS spectrum. This shows the presence of characteristic peaks of CS at 2924 and 2850 cm^−^^1^, which is not observed in the GEL–ALG spectrum, and a more intense band at 1641 and 1040 cm^−^^1^ which evidences the coupling of alginate, gelatin and CS signals.

Once the absorption of CS into the GEL–ALG scaffold was confirmed, it was important to study the stability of the GEL–ALG–CS scaffold. In order to assess this, we used metalloproteinase degradation assay at 37 °C for approximately 20 h. Figure 6C shows that after 5 h the GEL–ALG scaffold was completely degraded, whereas the GEL–ALG–CS scaffold remained for up to 20 h with 20% of its weight. These results suggest that the incorporation of *Cannabis sativa* oil could improve the stability of the hydrogels; this is an important property in a biomedical material. We evaluated the relative release kinetics of the different components of the *Cannabis sativa* oil extract: CBD, CBDA, CBN, THC and THCA. As it is shown in Figure 6D, approximately 15% is released after 24 h.

#### 3.2.1. Antioxidant Activity

**Figure 7:**
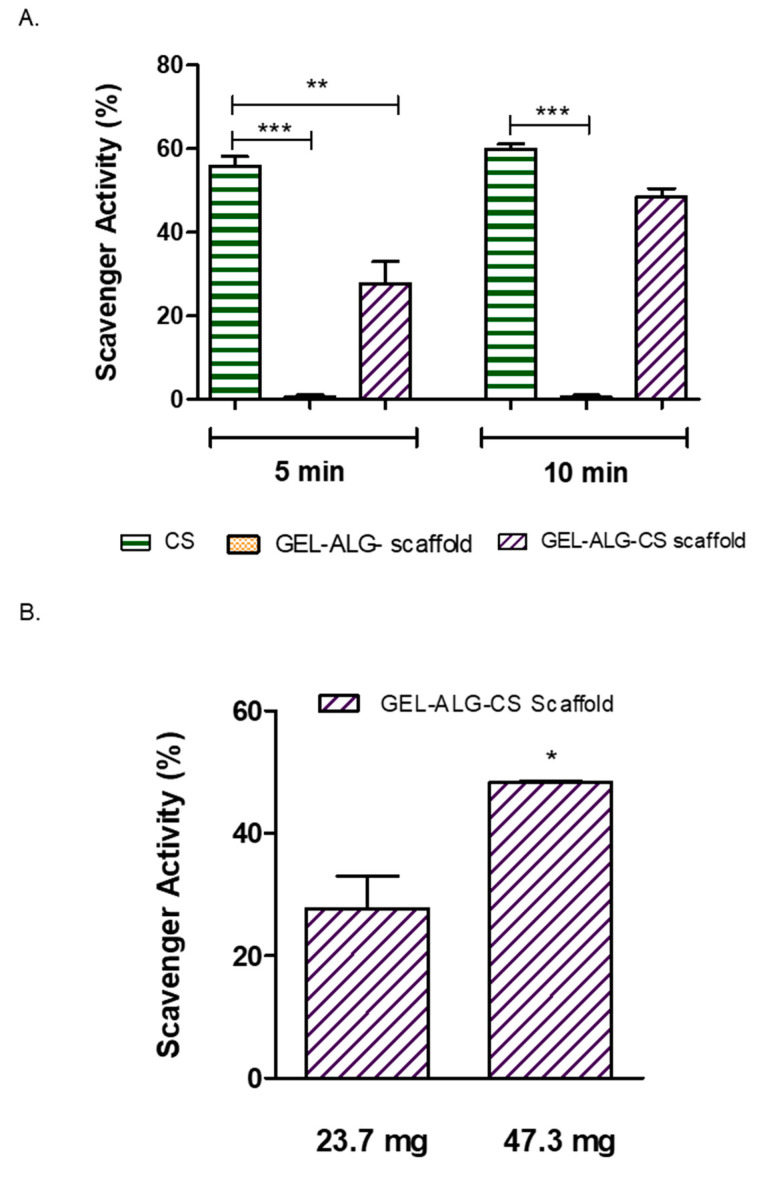
(**A**) Antioxidant activity of *Cannabis sativa* oil extract (CS), GEL–ALG scaffold and GEL–ALG–CS scaffold calculated as the % inhibition of DPPH radical at 5 and 10 min. (**B**) Antioxidant activity of GEL–ALG–CS scaffold calculated as the inhibition percentage of DPPH radical at different scaffold masses. Results are expressed as mean ± SD from triplicate experiments. * *p* < 0.05 ** *p* < 0.01 *** *p* < 0.001.

In order to accelerate the healing of chronic wounds, a variety of materials with antioxidant functions have emerged with the aim of removing the ROS excess to reduce oxidative stress, improve the wound microenvironment and ultimately achieve its rapid repair [62]. Our group has previously described the antioxidant capacity of collagen hydrogels loaded with *Cannabis sativa* oil extract [36], since it has a remarkable antioxidant capacity, stimulating wound healing [63]. Firstly, we studied the antioxidant activity at two different times (5 and 10 min) using the DPPH colorimetric assay. As it is shown in Figure 7A, CS extract oil presented a 60% scavenger activity at 5 min and the GEL–ALG–CS scaffold presented a 30% antioxidant activity. On the other hand, at 10 min, the CS extract oil remained at 60% antioxidant activity, whereas the GEL–ALG–CS scaffold increased its activity up to 50%. The increase in the antioxidant activity in the GEL–ALG–CS scaffold over time could be due to a controlled release of the CS extract oil. In addition, at 10 min there were no significant differences among CS extract oil and GEL–ALG–CS scaffold regarding the antioxidant activity. On the other hand, at both times the GEL–ALG scaffold did not have any activity, showing that the antioxidant activity is due to the *Cannabis sativa* oil.

Then, we decided to evaluate the difference in the antioxidant activity when changing the mass of the scaffold. In this sense, when we duplicated the mass there was an increase in the antioxidant activity, from 30% up to 50% in the bigger scaffold (Figure 7B). The latter allowed us to conclude that a higher mass of scaffold leads to the same activity being achieved at 10 min for the smaller scaffold.

#### 3.2.2. Antimicrobial Activity

**Figure 8:**
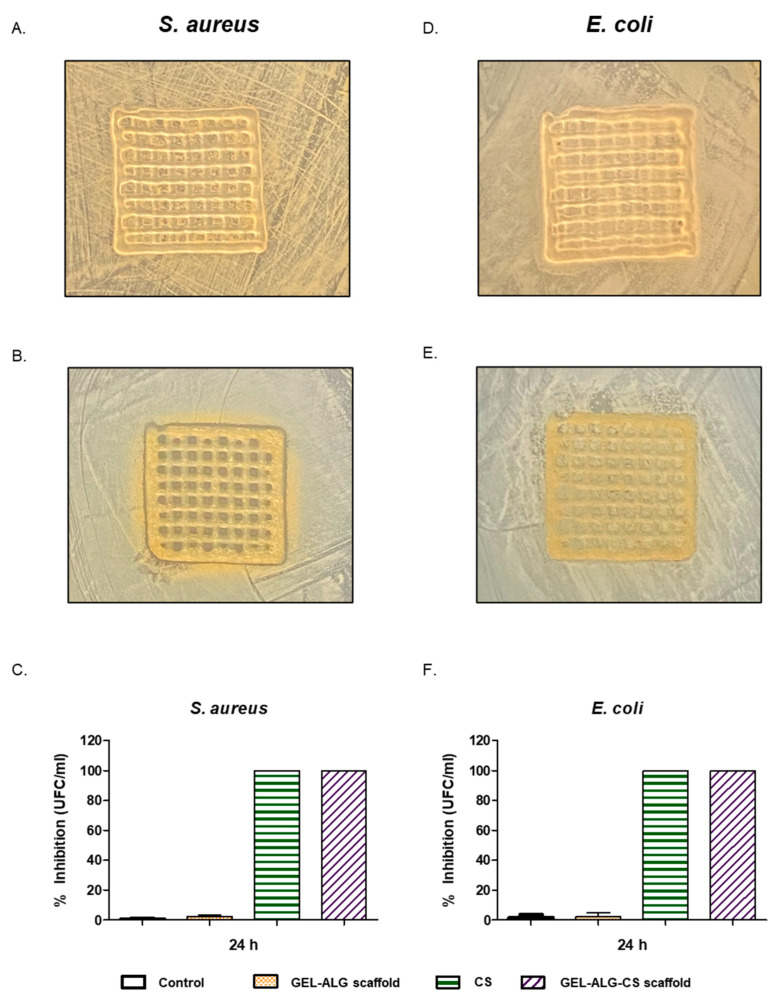
Antimicrobial activity: GEL–ALG scaffold (**A**) and GEL–ALG–CS scaffold (**B**) against *S. aureus* by disk diffusion method. (**C**) Percentage of growth inhibition of *S. aureus,* evaluated by the dilution method. GEL–ALG scaffold (**D**) and GEL–ALG–CS scaffold (**E**) against *E. coli* by disk diffusion method. (**F**) Percentage of growth inhibition of *E. coli,* evaluated by the dilution method.

It has been described that cannabinoids have antimicrobial activity against mainly Gram-positive bacteria and less against Gram-negative bacteria [39]. Therefore, we studied the antimicrobial activity of both the GEL–ALG scaffold and GEL–ALG–CS scaffold. When using the disk diffusion test, we found that the GEL–ALG–CS scaffold has antimicrobial activity, since there were inhibition zones around the scaffold and between the gaps inside (Figure 8B). This result suggests that the scaffold has antimicrobial activity against Gram-positive bacteria. On the other hand, the GEL–ALG–CS scaffold did not have any inhibition zones when it was exposed to Gram-negative bacteria, which means that *E. coli* grew in the presence of the scaffold (Figure 8E). This difference in antimicrobial activity among Gram-positive and Gram-negative bacteria could be attributed to the cannabidiols’ mechanism of action. In this vein, cannabidiols inhibit the synthesis of DNA, RNA, proteins and peptidoglycan and disturb the Gram-positive cytoplasmic membranes, with fewer actions in Gram-negative bacteria [40].

After that, we tested the antimicrobial activity of the scaffolds using the dilution method. Figure 8C,F show that the % inhibition of bacterial growth was greater than 99% for CS and GEL–ALG–CS scaffolds for both Gram-positive and Gram-negative bacteria. This result is consistent with a previous work of our group, where we showed a stronger bactericidal effect of the materials in the liquid medium than in the solid one [36]. These results show that CS has antimicrobial activity, and it is preserved when it is incorporated in the scaffolds.

#### 3.2.3. Biocompatibility of GEL–ALG–CS Scaffolds

**Figure 9:**
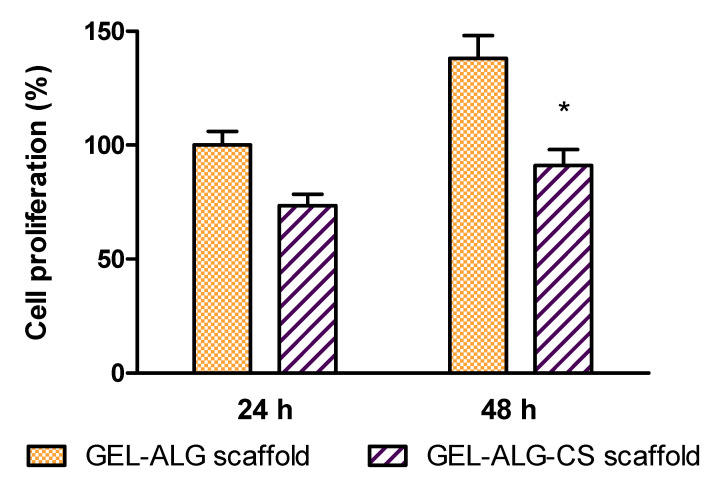
Viability of 3T3 fibroblasts evaluated by the MTT test at 24 h for GEL–ALG–CS scaffold. Viability rate in GEL–ALG scaffold was considered as 100%. Results are expressed as mean ± SD from triplicate experiments. * *p* < 0.05.

Cytotoxicity towards 3T3 fibroblast cells was also studied after incorporating CS into GEL–ALG scaffolds. After culturing fibroblasts in the presence of GEL–ALG–CS for 24 h, cell viability was measured by MTT assay. Figure 9 shows cell proliferation rate for GEL–ALG scaffolds and GEL–ALG scaffolds containing CS. Three-dimensional printed materials allow fibroblast cell proliferation. However, a weak reduction in cell growth rate was observed for hybrid scaffolds with cannabidiols adsorbed. It is well known that cell-biomaterial interactions are of paramount importance to support adhesion and proliferation [64]. Indeed, the formation of a layer on the surface of the scaffolds which may not favor cellular adhesion has been also reported [65]. Here, it can be proposed that the coating of phytocannabinoids on the surface of the scaffold results in hydrophobic surfaces that hinder the cellular adhesion and may therefore affect the proliferation of cells. Nevertheless, this decrease of cell proliferation in the presence of GEL–ALG–CS does not imply a significant growth inhibition [43,66], indicating that the 3D-printed material containing CS can be used as a biocompatible scaffold for wound healing. In addition, after 48 h the cells continued growing when exposed to both the GEL–ALG scaffold and GEL–ALG–CS scaffold.

## 4. Conclusions

In this work we have designed and developed a bioink made up of gelatin and alginate. The optimal conditions for its development were studied in depth, in search of the best conditions for printing the bioink in a 3D bioprinter. In this sense, it is extremely important to improve both the method of obtaining the bioink and the bioprinting parameters, since in this way it could be reproducible and scalable.

The scaffolds obtained have the versatility of being lyophilized for storage without losing their structure or integrity. The fact that they have a high absorption capacity could give rise to a potential use as scaffolding for the loading of different therapeutic molecules. Finally, the addition of *Cannabis sativa* oil contributed to the development of a new biomaterial with antioxidant and antimicrobial activity. In summary, herein we show that the new biomaterial loaded with *Cannabis sativa* oil and printed with 3D technology could be a promising alternative to conventional treatments for wound healing.

## Figures and Tables

**Table 1:** Lyophilization Parameters.

Freezing-Drying Step	Temperature (°C)	Time (h)	Pressure (mBar)
Freeze	−50	3	-
Vacuum chamber			0.2
Primary drying	−50 20	5 7	0.2 0.2
Secondary drying	20	24	-

**Table 2:** Different conditions during ink preparation.

Condition 1	Condition 2	Condition 3
Mix gelatin + alginate, then add PBS and finally stir at 60 °C for 2 h.	Mix gelatin + alginate, then add PBS and finally stir at room temperature for 2 h	First add gelatin to PBS, then add alginate in PBS, mix these solutions and finally stir at 60 °C for 2 h.

## Data Availability

The data presented in this study are available on request from the corresponding author.
